# Core-insulator embedded nanosheet field-effect transistor for suppressing device-to-device variations

**DOI:** 10.1038/s41598-024-58081-z

**Published:** 2024-03-29

**Authors:** Donghwi Son, Hyunwoo Lee, Hyunsoo Kim, Jae-Hyuk Ahn, Sungho Kim

**Affiliations:** 1 Department of Electrical Engineering, Sejong University, Seoul, 05006 Korea; 2 Department of Electronics Engineering, Chungnam National University, Daejeon, 34134 Korea; 3 Department of Electronic and Electrical Engineering, Ewha Womans University, Seoul, 03760 Korea

**Keywords:** Electrical and electronic engineering, Electronic devices

## Abstract

Nanosheet field-effect transistors (NSFETs) have attracted considerable attention for their potential to achieve improved performance and energy efficiency compared to traditional FinFETs. Here, we present a comprehensive investigation of core-insulator-embedded nanosheet field-effect transistors (C-NSFETs), focusing on their improved performance and device-to-device (D2D) variability compared to conventional NSFETs through three-dimensional device simulations. The C-NSFETs exhibit enhanced direct-current (DC) performance, characterized by a steeper subthreshold slope and reduced off-current, indicating better gate electrostatic controllability. Furthermore, the structural design of C-NSFETs enables to demonstrate a notable resilience against D2D variations in nanosheet thickness and doping concentration. In addition, we investigate the effects of interface traps in C-NSFETs, emphasizing the importance of thermal oxidation processes in the formation of core-insulating layers to maintain optimal device performance.

## Introduction

In the ongoing pursuit of advancements in semiconductor device technology aimed at augmenting the performance and minimizing the power consumption, diverse transistor structures have been investigated. Among these, nanosheet field-effect transistors (NSFETs) have emerged as promising alternatives to the conventional transistor structure, which consists of thin layers of channel material enclosed by horizontal gate electrodes^1,2^. An advantage of the NSFET is its superior electrostatic control over the channel with higher current drivability compared to other structures, such as nanowire-FETs^3–5^. Moreover, the performance of the NSFET can be easily adjusted by modifying the nanosheet width or stacking multiple nanosheets. Another critical advantage of NSFETs is their compatibility with the conventional FinFET fabrication processes. This compatibility, along with the enhanced performance metrics of NSFET, establishes them as an increasingly feasible alternative to FinFET technology, especially for advanced semiconductor technologies beyond the 5 nm node^1^.

However, as devices continue to scale down, NSFETs face challenges in sustaining improved performances. To address this issue, a structural modification involving the integration of an ultrathin SiGe shell layer within or around an Si nanosheet has been proposed^6–8^. These shell-type NSFETs exhibited improved DC performance and reduced negative-bias temperature instability, which was attributed to the modified energy band configuration. Additionally, the lattice mismatch between the nanosheet and shell induces strain effects, which consequently elevate the carrier mobility, contributing to improved on/off current ratios and subthreshold slopes. Despite these advancements, a comprehensive understanding of the shell-type NSFETs is crucial. A key concern is the device-to-device (D2D) variability, which primarily stems from fluctuations in nanosheet thickness, variations in shell layer dimensions, and inconsistencies in channel doping concentrations^9,10^. Additionally, interface traps resulting from defects at the nanosheet-shell interfaces are another source of variability. These traps can adversely affect device characteristics, including mobility degradation and threshold voltage instability^11–13^. Therefore, understanding the nature and impact of these D2D variabilities is imperative to improve the performance and reliability of advanced NSFETs.

Notably, an alternative structural design for NSFET, that is, core-insulator-embedded NSFET (C-NSFETs), has been proposed recently^14^. It incorporated a thin insulating layer within the nanosheet to reduce the leakage current relative to conventional NSFET. While the initial conception of C-NSFET highlighted its potential in improving DC performance metrics, several critical aspects of the C-NSFET remain unexplored. In this study, we aim to bridge this knowledge gap by presenting a comprehensive analysis of the C-NSFET, particularly focusing on its device-to-device (D2D) variability. We investigate the variations in nanosheet thickness, channel doping concentration, and the impact of interface traps on the performance of the C-NSFET. Our approach involves an examination of the additional fabrication processes required for the C-NSFET, such as oxidation and epitaxial silicon growth, and their potential to induce variabilities that could compromise device performance. By employing a three-dimensional device simulation, we demonstrate that the C-NSFET not only maintains enhanced DC performance but also exhibits improved resilience against D2D variations. These findings indicate that the C-NSFET represents a feasible pathway for further device scaling in semiconductor technology, potentially exceeding the capabilities of conventional NSFET, particularly in terms of reliability and stability.

## Result and discussion

### Device structure and simulation methodology

**Figure 1:**
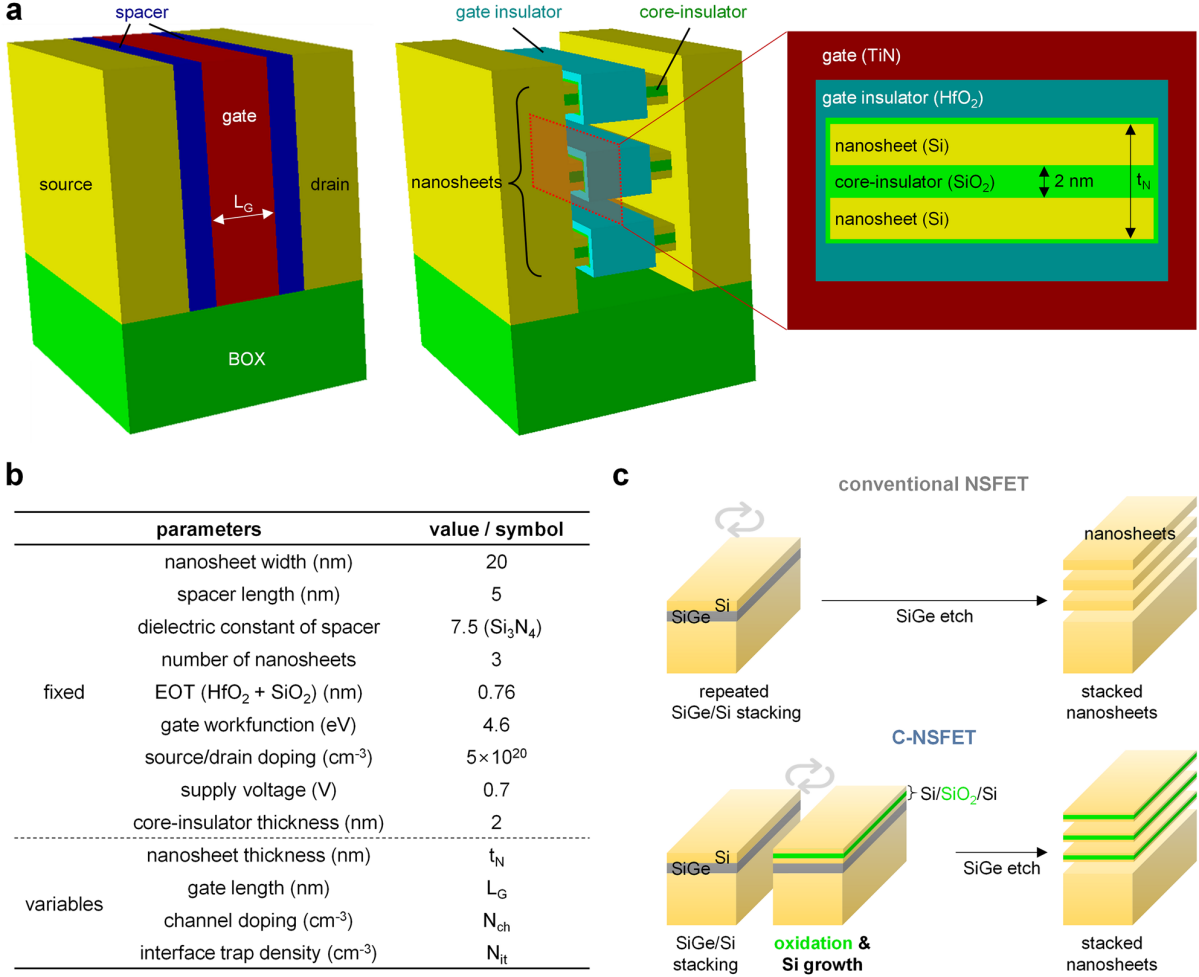
Device structure and parameters for C-NSFET. (**a**) Three-dimensional and cross-sectional views of the C-NSFET. (**b**) Design parameters of C-NSFET. (**c**) Fabrication process flow comparison between the conventional NSFET and C-NSFET.

Figure 1a presents three-dimensional schematics of the C-NSFET, accompanied by two-dimensional cross-sectional views. Figure 1b presents details of the design parameters, which conform with the International Technology Roadmap for Semiconductors (ITRS) guidelines for 5 nm technology node. In our analysis, subsequent parameters are held constant: nanosheet width (50 nm), spacer length (5 nm), number of nanosheets (3 nanosheets), equivalent oxide thickness (0.76 nm), workfunction of TiN gate (4.6 eV), source/drain doping concentrations (5 × 10^20^ cm^−3^), supply voltage (0.7 V, which is equivalent to the gate and drain voltages), and thickness of core-insulating layer (2 nm). The nanosheet thickness (t_N_), gate length (L_G_), channel doping concentration (N_ch_), and interface trap density (N_it_) were treated as variables in this study. Figure 1c shows a comparison of the fabrication process of the C-NSFET with that of a conventional NSFET. This process involves the selective etching of SiGe layers from cross-stacked SiGe/Si layers, which is the standard process for NSFET fabrication. Notably, the C-NSFET process incorporated additional thermal oxidation and Si growth steps, forming a core-insulating layer within the Si nanosheets. Here, thermal oxidation permits a minimized N_it_ at the interfaces between the Si nanosheet and SiO_2_ core-insulating layer, in the order of ~ 10^10^ cm^−2^^15^ Additionally, thermal oxidation enables precise control over the thickness of the core-insulating layer^16^, which is essential for mitigating D2D variations and will be further elucidated in subsequent discussions.

**Figure 2:**
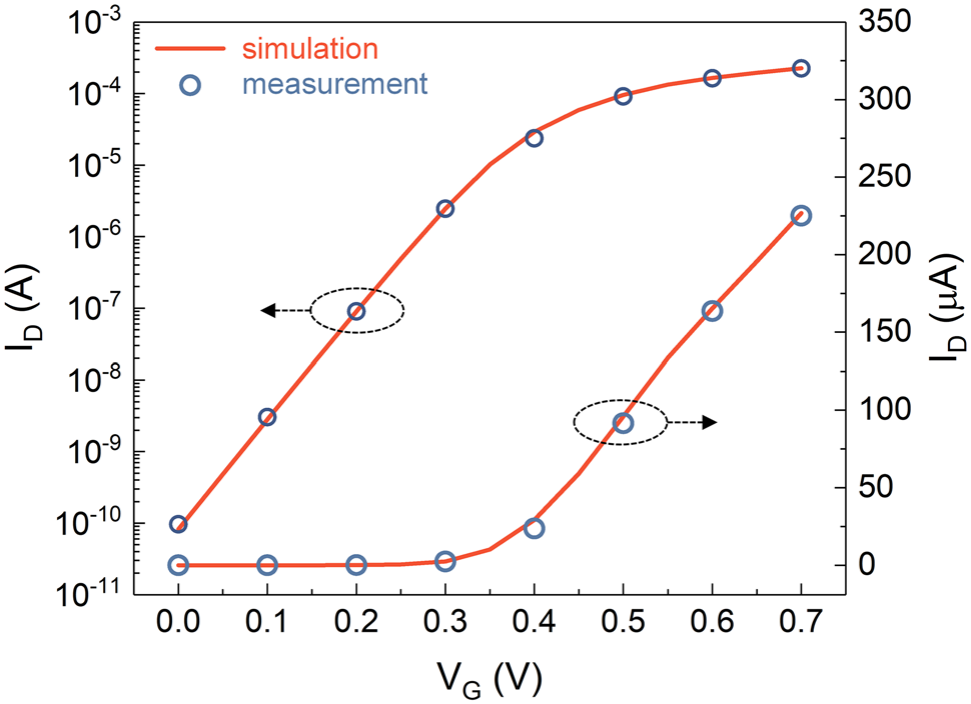
Validity of our simulation methodology. Calibration of simulation result with experimental in Ref.^1^.

All device simulations in this study were executed using the SILVACO ATLAS software. We employed a series of sophisticated models to align our simulations with empirical data. The Boltzmann transport equation enabled an accurate simulation of the carrier transport dynamics within the devices. Both the Shockley–Read–Hall model and trap-assisted Auger recombination models are crucial for evaluating the effects of carrier generation and recombination phenomena, particularly those associated with interface traps. In addition, Lombardi's mobility model accounts for various scattering mechanisms, including phonon/Coulomb and surface roughness scattering. Moreover, our simulations integrated field- and concentration-dependent mobility models to precisely capture any potential degradation in carrier mobility. To assess the influence of interface traps at the interfaces between the Si nanosheet and core-insulating layer, we included the observed characteristics of interface traps in Si/SiO_2_^17^ in our simulations. These trap energy levels were assigned to a single energy level located 0.2 eV away from both the conduction-band and valence-band edges. The capture cross-sections for electrons and holes were assigned as 10^−13^ and 10^−14^ cm^2^ respectively. Unless specifically analyzing the impact of changing N_it_, N_it_ was maintained at a fixed value of 10^10^ cm^−2^. To validate the robustness and accuracy of our simulation methodology, we compared our simulation with experimental data^1^, as depicted in Fig. 2. Our simulation results agree with the empirical data, demonstrating the effectiveness of our method in providing reliable and accurate predictions of the C-NSFET performance and behavior.

### DC performances

**Figure 3:**
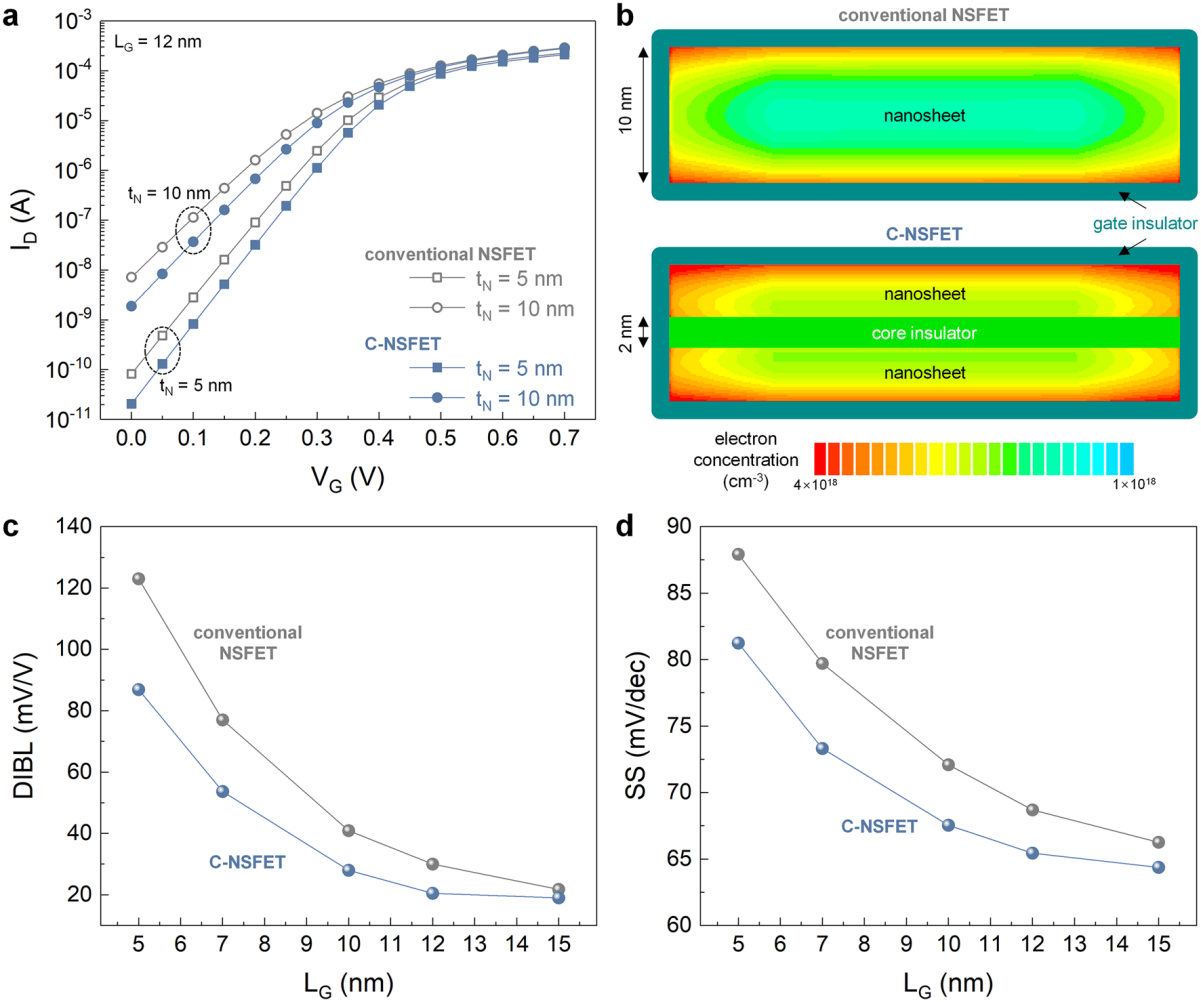
DC performance comparison. (**a**) Transfer characteristics of both the C-NSFET and conventional NSFET, in the case of t_N_ are 5 and 10 nm. Here, L_G_ and V_D_ are fixed to 12 nm and 0.7 V, respectively. (**b**) The cross-sectional contour map of electron concentration near the source junction, when both devices are fully on-state (V_G_ = V_D_ = 0.7 V). (**c**) DIBL and (d) SS characteristics of both devices, as a function of L_G_.

Figure 3a presents the transfer characteristics (i.e., drain current-gate voltage, I_D_–V_G_) for both the C-NSFET and conventional NSFET. Notably, the C-NSFET exhibited a steeper subthreshold slope and suppressed the off-current compared to the conventional NSFET, indicating enhanced gate electrostatic control in the C-NSFET. Figure 3b illustrates the electron density distribution near the source junction under the fully on-state condition (V_G_ = V_D_ = 0.7 V) for both device structures. The conventional NSFET exhibits a concentration of electron density predominantly near the nanosheet surface, with a notable decrease toward its core, indicating limited gate control over the core region of the nanosheet. In contrast, the C-NSFET has a more uniform electron density distribution across the nanosheet. This phenomenon is known as volume inversion, which is observable in an ultra-thin body^18,19^. Consequently, the C-NSFET enhanced the gate controllability over the entire nanosheet region, preventing short-channel effects (SCEs). Figure 3c and 3d further elaborate on this aspect by depicting the drain-induced barrier lowering (DIBL) and subthreshold slope (SS) behaviors as functions of the gate length (L_G_), providing a measure of the resistance of the devices to SCEs. The C-NSFET exhibits reduced DIBL and SS values compared to the conventional NSFET, confirming its superior gate controllability.

### Device-to-device variations

As discussed previously, D2D variations have become a critical concern in modern device technology. These variations arise from diverse sources, intricacies in fabrication processes, inherent properties of the materials used, and specific geometries of the devices. NSFETs with extremely scaled dimensions of approximately 5 nm for t_N_ and < 10 nm for L_G_ are vulnerable to D2D variations. Consequently, even minor deviations in these dimensions in the order of 1–2 nm, can have a significant impact on the device performance. Random dopant fluctuation (RDF) is another critical source of D2D variability in NSFETs. The inherently stochastic nature of ion implantation and diffusion processes in the fabrication of nanosheets can lead to inconsistencies in doping concentration and dopant placement within the channel region. Such variations directly influence the performance metrics of the NSFETs, including the threshold voltage, drive current, and power efficiency. Given these considerations, a comprehensive characterization of D2D variations is imperative to ensure reliable performance of NSFETs.

**Figure 4:**
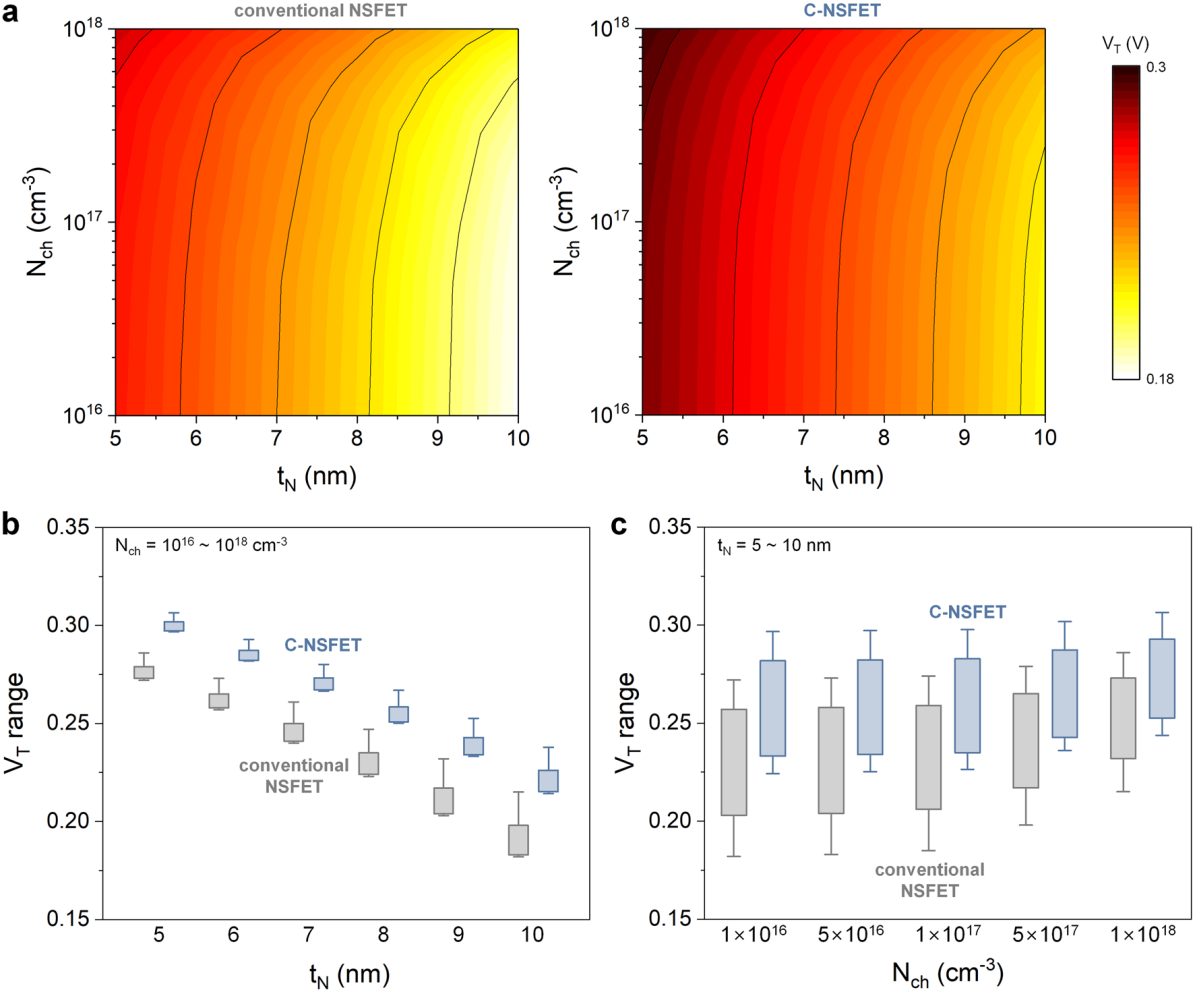
D2D variability of C-NSFET. (**a**) V_T_ values corresponding to the variations of N_ch_ and t_N_. The C-NSFET displays a slightly larger V_T_ than the conventional NSFET in all cases. Figure 4(**a**) is reconstructed to box-and-whisker plots, representing V_T_ variations in response to (**b**) fluctuations of N_ch_ (10^16^ ~ 10^18^ cm^−3^) with a fixed t_N_, and (**c**) fluctuations of t_N_ (5 ~ 10 nm) with a fixed N_ch_.

Figure 4a presents the simulated results of the threshold voltage (V_T_) for both the conventional NSFET and the C-NSFET, considering the variations in t_N_ and N_ch_. To analyze these results comprehensively, Fig. 4b and c were reconstructed into box-and-whisker plots, where the boxes represent the lower and upper quartiles, and the whiskers indicate the minimum and maximum values. Specifically, Fig. 4b focuses on the V_T_ variation in response to fluctuations in N_ch_ (ranging from 10^16^ to 10^18^ cm^−3^), under a fixed t_N_. This analysis aims to ascertain the impact of RDF on V_T_. Notably, the C-NSFET exhibits a smaller V_T_ variation than the conventional NSFET. This enhanced resistance to RDF can be attributed to the narrower nanosheet thickness of the C-NSFET. As already discussed in Fig. 3b, the higher gate controllability achieved from thinner nanosheets can suppress the V_T_ variation due to RDF, which is the same result studied previously in fully-depleted silicon-on-insulator (FDSOI) FETs^20–22^. This enhanced gate controllability results in a more uniform potential distribution in the channel region. Consequently, the influence of localized dopants is diminished. Moreover, volume inversion, that is, uniformity in the inversion charge distribution, also helps mitigate the local fluctuations in the potential caused by random dopants.

Additionally, Fig. 4c illustrates the V_T_ variation in response to fluctuations in t_N_ (ranging from 5–10 nm) while maintaining a constant N_ch_. The main focus of this analysis is to investigate the potential impact of the additional oxidation process in the C-NSFET, because fluctuations in t_N_ may occur during the formation of the core-insulating layer. Remarkably, the C-NSFET exhibits a V_T_ variation that was almost equivalent to that of a conventional NSFET. In the C-NSFET, the embedded core-insulating layer reduces the channel thickness, leading to a larger relative change in the channel thickness for the same absolute t_N_ variation. Nevertheless, both devices exhibit comparable V_T_ variations, suggesting that the D2D variation caused by oxidation in the C-NSFET is minimal. Consequently, the C-NSFET demonstrates enhanced resistance to D2D variations compared with the conventional NSFET in terms of fluctuations in t_N_ and N_ch_. Such stability is particularly crucial in advanced very-large-scale integration (VLSI) technology, in which the integration of a vast number of devices onto a single chip with high uniformity in device performance is required.

### The effects of interface traps

The emergence of traps at the interfaces between the nanosheets and core-insulating layers is a critical concern in the development of C-NSFETs. The incorporation of core-insulating layers within these nanosheets inherently introduces additional interfaces, potentially increasing N_it_ relative to that of conventional NSFET. The carrier scattering or trapping induced by these interface traps can detrimentally affect the device performance. To mitigate the generation of interface traps, we employed thermal oxidation to form the core-insulating layer in the C-NSFET, maintaining N_it_ of 10^10^ cm^−2^^17^ However, thermal oxidation did not prevent the increase in the number of interface traps along the sidewalls of the nanosheet^23^. Moreover, the utilization of high-k dielectrics for the core-insulating layer can increase N_it_ to approximately 10^12^ cm^−2^^24^. Consequently, a thorough understanding of the impact of these interface traps is imperative to ensure the reliability and consistency of C-NSFET performance.

**Figure 5:**
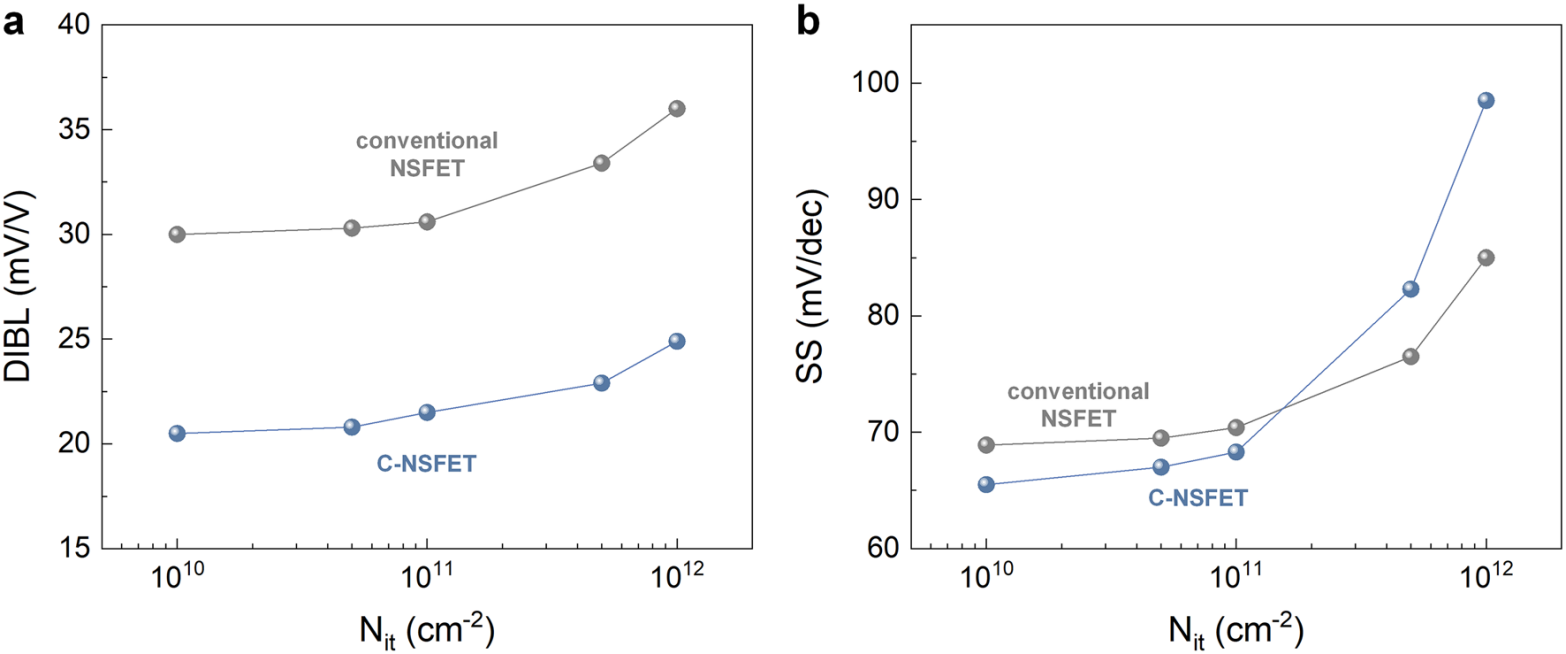
Effects of interface traps. (**a**) DIBL and (**b**) SS characteristics in both devices, as a function of N_it_.

Figure 5a and 5b show the DIBL and SS behaviors in both the conventional NSFET and the C-NSFET as a function of N_it_. As already discussed in Fig. 3c and d, the C-NSFET shows lower DIBL and SS values than the conventional NSFET, with a low N_it_ (10^10^ cm^−2^). When N_it_ is increased to 10^11^ cm^−2^, noticeable differences could not be observed in DIBL and SS values compared to the 10^10^ cm^−2^ level. However, with a further increase in N_it_ to 10^12^ cm^−2^, both the DIBL and SS values were significantly degraded. These findings indicate that the thermal oxidation process is critical in forming the core-insulating layer to minimize N_it_ below 10^11^ cm^−2^. Notably, the C-NSFET exhibits higher SS values than the conventional NSFET when N_it_ exceeds 10^11^ cm^−2^. Therefore, the utilization of alternative high-k dielectrics for the core insulating layer may contribute to higher N_it_ levels, potentially degrading the performance of C-NSFETs.

## Conclusions

In this study, we comprehensively evaluated the C-NSFET, focusing on its enhanced performance and immunity against D2D variations. The C-NSFET exhibited superior gate electrostatic control, as evidenced by a steeper subthreshold slope and reduced off-current, ensuring improved short-channel effect prevention. Additionally, the C-NSFET exhibits notable resilience to D2D variability, where the minimized impact of the RDF and stability against nanosheet thickness variations are pivotal in ensuring consistent device performance. Additionally, the strategic use of thermal oxidation to form a core-insulating layer effectively controls the N_it_. Despite the inherent increase in N_it_ owing to the additional interfaces in the C-NSFET structure, this does not significantly degrade the device performance, especially when compared with conventional NSFETs. Therefore, the C-NSFET has emerged not only as a feasible but also as a superior alternative for next-generation semiconductor device technologies, offering a combination of enhanced performance and reduced variability, which are essential for progression beyond the 5 nm technology node.

## Data Availability

The datasets used and/or analyzed during the current study available from the corresponding author on reasonable request.

## References

[1] Loubet, N. *et al.* Stacked nanosheet gate-all-around transistor to enable scaling beyond FinFET. In *Digest of Technical Papers—Symposium on VLSI Technology* (2017).

[2] Veloso A (2020). Nanowire & nanosheet FETs for ultra-scaled, high-density logic and memory applications. Solid State Electron..

[3] Sreenivasulu VB, Narendar V (2022). Design insights of nanosheet FET and CMOS circuit applications at 5-nm technology node. IEEE Trans. Electron Devices.

[4] Ajayan J (2021). Nanosheet field effect transistors-a next generation device to keep Moore’s law alive: An intensive study. Microelectron. J..

[5] Nagy D (2020). Benchmarking of FinFET, nanosheet, and nanowire FET architectures for future technology nodes. IEEE Access.

[6] Xu H (2023). Physical insights of Si-core-SiGe-shell gate-all-around nanosheet pFET for 3 nm technology node. IEEE Trans. Electron Devices.

[7] Cheng, S. L., Lv, S. S., Li, C., Dong, X. Y. & You, H. L. 2022 Investigate on DC characteristics and NBTI of SiGe core-shell nanosheet FET. In *International Conference on Solid-State and Integrated Circuit Technology (ICSICT) 2022* (2022).

[8] Barik R, Dhar RS, Awwad F, Hussein MI (2023). Evolution of type-II hetero-strain cylindrical-gate-all-around nanowire FET for exploration and analysis of enriched performances. Sci. Rep..

[9] Harsha Vardhan P, Amita G, S. & Ganguly, U. (2019). Threshold voltage variability in nanosheet GAA transistors. IEEE Trans. Electron Devices.

[10] Yoon JS (2022). DC performance variations by grain boundary in source/drain epitaxy of sub-3-nm nanosheet field-effect transistors. IEEE Access.

[11] Rathore S, Kumar Jaisawal R, Kondekar PN, Bagga N (2023). Trap and self-heating effect based reliability analysis to reveal early aging effect in nanosheet FET. Solid State Electron..

[12] Samadder T (2021). A physical model for bulk gate insulator trap generation during bias-temperature stress in differently processed p-channel FETs. IEEE Trans. Electron Devices.

[13] Chen W (2019). Investigation of PBTI degradation in nanosheet nFETs with HfO_2_ gate dielectric by 3D-KMC method. IEEE Trans. Nanotechnol..

[14] Joung, S. & Kim, S. Leakage performance improvement in multi-bridge-channel field effect transistor (MBCFET) by adding core insulator layer. In *International Conference on Simulation of Semiconductor Processes and Devices (SISPAD)* (2019).

[15] Reed ML, Plummer JD (1988). Chemistry of Si-SiO_2_ interface trap annealing. J. Appl. Phys..

[16] Miyazaki S, Nishimura H, Fukuda M, Ley L, Ristein J (1997). Structure and electronic states of ultrathin SiO_2_ thermally grown on Si(100) and Si(111) surfaces. Appl. Surf. Sci..

[17] Schulz M (1983). Interface states at the SiO_2_-Si interface. Surf. Sci..

[18] Venkatesan S, Neudeck GW, Pierret RF (1992). Dual-gate operation and volume inversion in n-channel SOI MOSFET’s. IEEE Electron Device Lett..

[19] Uchida, K., Koga, J. & Takagi, S. I. Experimental study on carrier transport mechanisms in double- and single-gate ultrathin-body MOSFETs—Coulomb scattering, volume inversion, and δT SOI-induced scattering. In *International Electron Devices Meeting (IEDM)* 805–808 (2003).

[20] Ohtou T, Sugii N, Hiramoto T (2007). Impact of parameter variations and random dopant fluctuations on short-channel fully depleted SOI MOSFETs with extremely thin BOX. IEEE Electron Device Lett..

[21] Rao R, Dasgupta N, Dasgupta A (2010). Study of random dopant fluctuation effects in FD-SOI MOSFET using analytical threshold voltage model. IEEE Trans. Device Mater. Reliab..

[22] Markov S, Cheng B, Asenov A (2021). Statistical variability in fully depleted SOI MOSFETs due to random dopant fluctuations in the source and drain extensions. IEEE Electron Device Lett..

[23] Lee K (2021). Defect spectroscopy of sidewall interfaces in gate-all-around silicon nanosheet FET. Nanotechnology.

[24] Wong H, Zhan N, Ng KL, Poon MC, Kok CW (2004). Interface and oxide traps in high-κ hafnium oxide films. Thin Solid Films.

